# Patterns of Anthracycline-Based Chemotherapy-Induced Adverse Drug Reactions and Their Impact on Relative Dose Intensity among Women with Breast Cancer in Ethiopia: A Prospective Observational Study

**DOI:** 10.1155/2020/2636514

**Published:** 2020-02-21

**Authors:** Diriba Alemayehu Gadisa, Mathewos Assefa, Gosaye Mekonen Tefera, Getnet Yimer

**Affiliations:** ^1^College of Medicine and Health Sciences, Pharmacy Department, Ambo University, Ambo, Ethiopia; ^2^School of Medicine, College of Health Sciences, Radiotherapy Center, Addis Ababa University, Addis Ababa, Ethiopia; ^3^Ohio State Global One Health Initiative, Office of International Affairs, The Ohio State University, Addis Ababa, Ethiopia

## Abstract

**Background:**

The breast cancer chemotherapy leads to diverse aspects of noxious or unintended adverse drug reactions (ADRs) that cause the relative dose intensity (RDI) reduced to below optimal (i.e., if the percentage of actual dose received per unit time divided by planned dose per unit time is less than 85%). Hence, this prospective observational study was conducted to evaluate chemotherapy-induced ADRs and their impact on relative dose intensity among women with breast cancer in Ethiopia.

**Methods:**

The study was conducted with a cohort of 146 patients from January 1 to September 30, 2017, Gregorian Calendar (GC) at the only nationwide oncology center, Tikur Anbessa Specialized Hospital (TASH), Addis Ababa, Ethiopia. The ADRs of the chemotherapy were collected using the National Cancer Institute (NCI) Common Terminology Criteria for Adverse Events (CTCAE) (version 4.03). The patients were personally interviewed for subjective toxicities, and laboratory results and supportive measures were recorded at each cycle. SPSS version 22 was used for analysis.

**Results:**

Grade 3 neutropenia (23 (15.8%)) was the most frequently reported ADR among grade 3 hematological toxicity on cycle 4. However, overall grade fatigue (136 (93.2%)) and grade 3 nausea (31 (21.2%)) were the most frequently reported nonhematological toxicities on cycle 1. The majority of ADRs were reported during the first four cycles except for peripheral neuropathy. Oral antibiotics and G-CSF use (17 (11.6%)) and treatment delay (31 (21.2%)) were frequently reported on cycle 3. Overall, 61 (41.8%) and 42 (28.8%) of study participants experienced dose delay and used G-CSF, respectively, at least once during their enrollment. Of the 933 interventions observed, 95 (10%) cycles were delayed due to toxicities in which neutropenia attributed to the delay of 89 cycles. Forty-four (30.1%) of the patients received overall RDI < 85%. Pretreatment hematological counts were significant predictors (*P* < 0.05) for the incidence of first cycle hematological toxicities such as neutropenia, anemia, and leukopenia and nonhematological toxicities like vomiting.

**Conclusion:**

Ethiopian women with breast cancer on anthracycline-based AC and AC-T chemotherapy predominantly experienced grade 1 to 3 hematological and nonhematological ADRs, particularly during the first four cycles. Neutropenia was the only toxicity that led to RDI < 85%. Thus, enhancing the utilization of G-CSF and other supportive measures will improve RDI to above 85%.

## 1. Background

Treatment of breast cancer by chemotherapy significantly increases disease-free survival (DFS) [1, 2] and overall survival (OS) [2]. However, in addition to damaging cancer cells, it also damages healthy cells which leads to diverse aspects of noxious and unintended reactions called adverse drug reactions (ADRs) [3, 4].

Most of the common and severe types of chemotherapy ADRs have been reported from clinical trials in which those at risk of complications are often excluded and safety monitoring may be more intensive than routine clinical care [3]. Moreover, subjective toxicities are at high risk of underreporting by physicians, which strongly supports the incorporation of patient-reported outcomes into toxicity reporting [5, 6].

A large number of individuals (i.e., >86%) undergoing chemotherapy in the United State of America (USA) and Australia reported at least one ADR during their cancer treatment [3, 7]. And most of the chemotherapy-related ADRs affect a diverse aspect of patient's quality of life [7] and further impair optimum chemotherapy delivery/relative dose intensity (RDI) [8] (i.e., the amount of drug administered per unit time expressed as a percentage of the planned dose) [9].

The study indicated that regimens consisting of cyclophosphamide and doxorubicin (Adriamycin) had significantly higher rates of chemotherapy-related complications [10] that lead to reduced RDI [8]. RDI is a significant predictor of survival in patients with cancer [8, 11]. Dose reductions and treatment delays that lead to reduced RDI could be minimized by utilizing prophylactic colony-stimulating factors (CSFs) and educating patients about the importance of adhering to their treatment schedule [11, 12].

Nevertheless, the importance of maintaining relative dose intensity (RDI) is well known; little information is available from routine clinical practice regarding how well dose intensity is maintained with modern chemotherapy regimens [13].

Patient-reported outcomes (PROs) are already considered as the gold standard for data collection in closely related research areas, including assessment of health-related quality of life (HRQoL), treatment preferences, and satisfaction with care [14]. Consequently, the National Cancer Institute (NCI) Common Terminology Criteria for Adverse Events (CTCAE) (version 4.03), with its PRO part for subjective toxicities, were used to collect the toxicity information [15, 16].

The objective of this study was to evaluate the frequency and severity of ADRs due to anthracycline-based chemotherapy (i.e., Adriamycin-Cyclophosphamide (AC) and Adriamycin-Cyclophosphamide followed by paclitaxel (AC-T)) and their impact on RDI among Ethiopian women with breast cancer in routine clinical care. Also, it aimed to assess patterns of supportive measures given during chemotherapy courses which are largely unknown.

## 2. Methods

The institutional-based prospective cohort study was conducted from January 1 to September 30, 2017, Gregorian Calendar (GC) at the only nationwide oncology center, Tikur Anbessa Specialized Hospital (TASH), Addis Ababa, Ethiopia.

### 2.1. Inclusion Criteria

- Women of age above 18 years with proven newly diagnosed breast cancer (i.e., stage I to IV).
- Patients scheduled to receive the most commonly used neo/adjuvant or palliative first-line chemotherapy (i.e., Adriamycin-cyclophosphamide (AC) and Adriamycin-cyclophosphamide followed by paclitaxel (AC-T) regimen).
- Patients with no missing data during the cohort.

### 2.2. Exclusion Criteria

- Patients who had previously received breast cancer treatment (i.e., currently on the second line for recurrent breast cancer).
- Patients with psychiatric disorders and other severe medical illnesses.

Sample size (*N*) was calculated based on single proportion formula [*N* = *Z*^2∗^*P* (1 − *P*)/*d*^2^] with 0.05 margins of errors (d), 95% confidence interval (*Z* = 1.96). And the prevalence (*P*) of at least one chemotherapy ADR in breast cancer patients was 93% [17]. Hence, 101 patients are at least required. However, for the robustness of the study, we enrolled more study participants (*N* = 146).

### 2.3. Study Variables

Baseline demographic and clinical variables included age and performance status; body surface area (BSA), cancer stage, comorbidities, and planned chemotherapy treatment were collected at the baseline/pretreatment level. Also, data on chemotherapy drugs, schedule and dosing information, routine laboratory tests, G-CSF use, dose delay, oral antibiotic use, and adverse drug reactions were collected at each cycle. The information about adverse drug reactions during the prior cycle was collected at the beginning of the next cycle.

### 2.4. Study Outcomes

The primary endpoints of the study were chemotherapy-associated adverse drug reactions. Secondary endpoints included supportive care to reduce the chemotherapy-associated toxicities, such as dose delay, use of G-CSF and antibiotics, and reductions in chemotherapy RDI.

### 2.5. Statistical Methods

The proportions were presented for all relevant clinical categorical variables. The continuous variables were evaluated using standard measures of central tendency and variability summarized via descriptive statistics such as mean and standard deviation. The proportions of patients with adverse drug events and the proportions of patients receiving supportive interventions were calculated by cycle and cumulatively across all cycles. SPSS version 22.0 was used for analysis. The model fitness for the variables was evaluated by the Hosmer–Lemeshow goodness of fit test; Hosmer–Lemeshow goodness of fit tests with *P* value ≥0.22 was used for the model fitness of multivariate backward binary logistic regression analysis. To identify determinants of most frequent first cycle ADR, multiple stepwise backward logistic regression analysis was done, and statistical significance was considered at *P* value ≤0.05. Written informed consent was sought and data were secured.

### 2.6. Treatment Modalities

Forty-six (31.5%) and 25 (17.1%) women with breast cancer on AC regimen received doxorubicin (A) 60 mg/m^2^ and cyclophosphamide (C) 600 mg/m^2^ as an intravenous infusion repeated every 21 days for four and six cycles, respectively, while 75 (51.4%) study participants on an AC-T regimen received doxorubicin (A) 60 mg/m^2^ and cyclophosphamide (C) 600 mg/m^2^ for four cycles and followed by paclitaxel (T) 175 mg/m^2^ intravenous infusion repeated every 21 days for 4 cycles.

Besides, for every cycle of treatment, premedication with ondansetron 8 mg, dexamethasone 16 mg, cimetidine 400 mg, and metoclopramide 10 mg was given by intravenous infusion before the commencement of chemotherapy.

### 2.7. Assessment for Safety Endpoints

The standard approach to adverse events (AEs) reporting in cancer clinical research is the Common Terminology Criteria for Adverse Events (CTCAE), which is maintained by the USA National Cancer Institute (NCI). The CTCAE (version 4.03) consist of 790 individual items, with 78 symptomatic AEs which are amenable for self-reporting by patients (i.e., patient-reported outcome/PRO version), each representing a discrete event which is graded for severity on a five-point scale based on clinical criteria. There are three general categories of AEs in the CTCAE: laboratory-based events (e.g., neutropenia), observable/measurable events (e.g., retinal tear), and symptomatic adverse events (e.g., nausea). Each of these is graded as 1 (mild), 2 (moderate), 3 (severe), 4 (life-threatening), or 5 (death) according to an internationally agreed standard (Common Terminology Criteria for Adverse Events) [15, 16]. Hence, we used the NCI CTCAE measurement system (version 4.03) to code for the toxicities related to AC and AC-T regimen chemotherapy. The patients were personally interviewed for subjective toxicities such as nausea, vomiting, and the like, and their toxicity grades were assessed based on the diary maintained during their revisits. We made an interview for those symptomatic/subjective toxicities using the two most popular languages in Ethiopia, Amharic and Afaan Oromoo, after backward-forward translation by two bilingual oncology nurses and one principal investigator. We presented the detail of each AE used in our study, which was abstracted from NCI CTCAEs (version 4.03), in the supplementary material ([Supplementary-material supplementary-material-1]).

### 2.8. Relative Dose Intensity (RDI) Determination

  First step: planned (standard) dose intensity of each drug

(1) $$\begin{array}{c} \frac{\text{Planned full dose of the drug per cycle }\left({\text{mg/m}}^{2}\right)}{\text{Planned number of weeks in cycle }\left(\text{week}\right)}. \end{array}$$

  Second step: actual dose intensity for each drug

(2) $$\begin{array}{c} \frac{\text{The total dose of the drug actually received by the patient }\left({\text{mg/m}}^{2}\right)}{\text{Total number of weeks actually needed to receive a total dose }\left(\text{week}\right)}. \end{array}$$

  Third step: RDI (%) of each drug

(3) $$\begin{array}{c} \frac{\text{Actual dose intensity of each drug}}{\text{Planned dose intensity of each drug}}\times 100\text{.} \end{array}$$

  Source of the formula: Pettengell et al. [18].

## 3. Results

### 3.1. Sociodemographic and Clinical Characteristics of Study Participants

The study participants had 42.2 (±11.5) years and 1.6 (±0.19) m^2^ mean age and body surface area, respectively. The majority of the study participants had stage III (64 (43.8%)) and ECOG performance I (135 (92.5%)). The means of all laboratory values were within the normal range. The study participants received a median of 8 cycles of chemotherapy (Table 1).

### 3.2. Overall Grade Toxicity Profile, the Pattern of G-CSF Use, and Treatment Delay at Each Cycle of Chemotherapy among the Study Participants

The highest frequencies of hematological toxicities were recorded at 4^th^ cycles of chemotherapy, including overall grade leucopenia (68 (46.6%)), neutropenia (59 (40.4%)), anemia (31 (21.2%)), and thrombocytopenia (5 (3.4%)). The most frequent grade 3 hematological toxicities were reported during cycles 3 and 4. Overall grade fatigue (136 (93.2%)), nausea (124 (85%)), vomiting (96 (65.8%)), and oral mucositis (53 (36.3%)) were the most frequently reported nonhematological toxicities during the first four cycles. However, peripheral neuropathy was frequently reported during the 5^th^ to 8^th^ cycles of chemotherapy (Table 2). Neutropenia during the first six cycles (see row 5 of Table 2) and infection on 5^th^ cycle (see row 15 column 6) were the only two grade 4 toxicities reported during the cohort.

The highest frequency of G-CSF use (11.6% on cycle 3 and 10.3% on cycle 4) and treatment delay (21.2% on cycle 3 and 14.4% on cycle 4) were reported (see columns 3 and 4 with rows 22 to 24 in Table 2).

### 3.3. The Pattern of Cumulative G-CSF Use, Dose Delay, and Toxicities Related to Dose Delay

**Figure 1:**
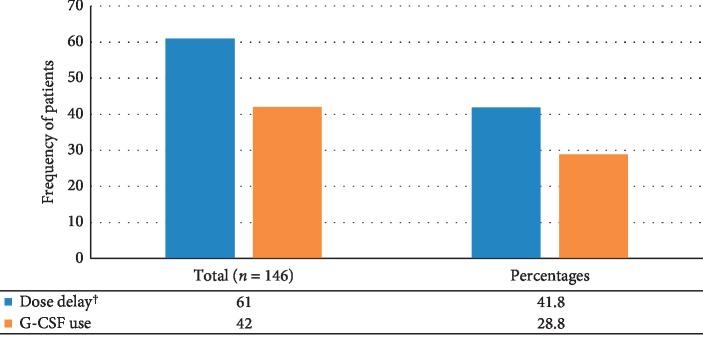
Overall incidence of dose delay and pattern of G-CSF use among women with breast cancer on AC and AC-T chemotherapy, from January 1 to September 30, 2017 GC, *N* = 146. ^†^The median for dose delay was seven days.

**Figure 2:**
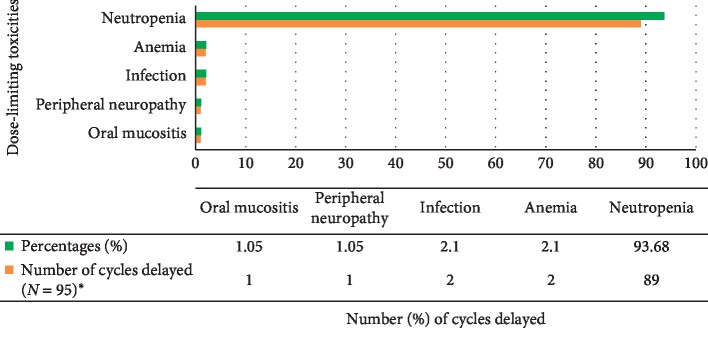
Toxicities related to dose delay among women breast cancer patients on AC and AC–T chemotherapy, from January 1 to September 30, 2017 GC, *N* = 95. ^*∗*^933 cycles were delivered for 146 patients, in which 95 (10%) cycles were delayed due to toxicities.

Dose delay, at least once, due to chemotherapy toxicities has happened in 61 (41.8%) of the study participants. Forty-two (28.8%) of study participants used G-CSF at least once during their chemotherapy courses to treat chemotherapy-induced neutropenia (Figure 1). Ninety-five cycles were delayed due to chemotherapy toxicities in which neutropenia contributed to the delay of eighty-nine (93.68%) cycles (Figure 2).

### 3.4. Relative Dose Intensity of Adriamycin (A), Cyclophosphamide (C), and Paclitaxel (T)

The relative dose intensity (RDI) of Adriamycin, cyclophosphamide, and paclitaxel was 90.4%, 90.4%, and 93.4%, respectively. 38/146 (26%) of study participants had Adriamycin's and cyclophosphamide's RDI of less than 85%. Conversely, among 75 patients who received paclitaxel, 9 (12%) had RDI less than 85%. Considering RDI's of all agents together, 102 (69.9%) of study participants had RDI ≥ 85%, i.e., 44 (30.1%) had RDI < 85% (Table 3).

### 3.5. Determinants of Overall RDI ≥ 85% among the Study Participants

Grade ¾ neutropenia (AOR = 0.26, *P*=0.001) and receiving 8 cycles (AOR = 0.28, *P*=0.006) were the two significant determinants that decrease the probability of having overall RDI ≥ 85% by 74% and 72%, respectively (Table 4).

### 3.6. Factors Associated with the Incidence of First Cycle Toxicity

Lower baseline neutrophil counts (AOR = 0.670, *P*=0.002) were the only significant predicting factor for the occurrence of first cycle neutropenia. Patients with lower baseline hemoglobin (AOR = 0.735, *P*=0.044) and white blood cells count (AOR = 0.718, *P*=0.002) were more likely to suffer from the first cycle leukopenia. Conversely, patients with lower baseline hemoglobin (AOR = 0.339, *P* ≤ 0.001) and higher baseline neutrophil counts (AOR = 1.332, *P* ≤ 0.001) were more likely to experience first cycle anemia (see Table 5). Younger age (AOR = 0.964; *P*=0.021), lower baseline WBC (AOR = 0.856; *P*=0.047), and lower baseline hemoglobin (AOR = 0.700, *P*=0.016*P* = 0.016) were significant predictors for those who experienced vomiting on the first cycle (Table 5).

## 4. Discussion

Due to treatment effects on nonneoplastic cells, severe physical, emotional, and cognitive treatment-related symptoms may appear during or shortly after the delivery of chemotherapy [19]. As a result, our finding indicates that the majority of the toxicities were reported during the first four cycles of chemotherapy. And during these cycles, all of our study participants received only Adriamycin and cyclophosphamide from both regimens. Recent systematic review and meta-analysis indicated that both hematological (i.e., neutropenia and others) and nonhematological toxicities (i.e., nausea, vomiting, and mucositis) were common in anthracycline (Adriamycin) containing regimen for breast cancer [20].

The epithelium covering the entire gastrointestinal tract (GIT) is rapidly dividing, and thus it is highly susceptible to chemotherapy which leads to probably the most feared adverse effects, nausea and vomiting [21]. The most frequent GIT adverse effects were reported during the first four cycles of chemotherapy by our study participants. The most frequent nausea (124 (85%)) and vomiting (96 (68.5%)) were reported on the 3^rd^ cycle. However, 53 (36.3%) of the patients reported oral mucositis on the second cycle. On top of that, the most frequent grade 3 nausea (31 (21.2%)) was reported on the first cycle while the most frequent grade 3 vomiting (14 (9.6%)) and oral mucositis (9 (6.2%)) were recorded on 4^th^ cycle. On contrary to the first four cycles, the incidence of chemotherapy-induced GIT adverse effects became less frequent in particular during cycles 7 and 8. Only five study participants complained of nausea and eight complained of oral mucositis while none of them reported grade 3 and above during the last two cycles. In line with Rašić et al.'s finding [22], the majority of our study participants experienced grade ½ GIT toxicities.

On contrary to the GIT adverse effects, the incidence and severity of peripheral neuropathy were increased through cycle 5 to cycle 8. Of 75 study participants in the present study, 64 (85.3%) of them complained overall grade peripheral neuropathy and 15 (20%) reported grade 3 peripheral neuropathy on the 7^th^ cycle. It happened because the study participants on AC-T regimen (*N* = 75) started to receive paclitaxel after 4 cycles of AC. And it is known that paclitaxel is frequently associated with neurotoxicity [22, 23]. However, the incidence and severity of other toxicities were decreased after the patients begun paclitaxel. This supports the evidence of adding paclitaxel seqeuntially to the anthracycline-based regimen does not increase in overall incidence and severity of the toxicity [22]. Moreover, it also strengthens the fact that the standard dose of paclitaxel causes less frequent GIT and hematological adverse effects than neurotoxicity [24–26].

The other frequent nonhematological toxicity reported in our study was fatigue, with the highest frequency on cycle 1. However, the incidence of fatigue was decreased from 1^st^ cycle (136 (93.2%)) to 8^th^ cycle (49 (66.2%)) with the most frequent grade 3 on 4^th^ cycle (6 (4.1%)). Studies reported that fatigue shows a high and fluctuating prevalence similar to a roller-coaster pattern during treatment with chemotherapy [3, 19], and mostly it occurred independently of any anemia [21]. Recommended physical activity levels are suggested to decrease this debilitating fatigue [19].

The study conducted on patients with breast and other cancers in the USA revealed that fatigue was the most frequent (88%) and adverse effect reported while nausea/vomiting was 48% [7]. Furthermore, a pooled analysis of randomized controlled trials reported different grade ¾ toxicities such as nausea (3.1%), vomiting (1.9%), mucositis (2.4%), and fatigue (5.3%), and which frequently occurred during treatment with the regimen containing Adriamycin and cyclophosphamide (*P* < 0.0026) [23]. The incidences of these chemotherapy-induced nonhematological toxicities reported by our study participants were higher than those of the results reported in a clinical trial [27]. This perhaps is due to underreporting of toxicities by physicians and/or due to the nature of randomized controlled trials (RCTs) where enrollment criteria are strict [5, 6].

Unfortunately, no grade 3 and above serum creatinine increment was recorded during chemotherapy which indicates that the renal toxicity of AC/AC-T regimen was about only grade ½ with maximum counts on the 3^rd^ cycle (13 (8.9%)). The most frequent AST/ALT 11 (11%) and ALP 18 (18%) increment were reported on the 5^th^ cycle. Two (1.4%) patients had grade 3 AST/ALT and/or ALP increment during the first two cycles of treatment. However, women with nonmetastatic breast cancer treated with the AC-T regimen in Korea experienced a less frequent increase in ALP 0 (0%) and creatinine 1 (2.9%) compared with our finding though they had an increased AST/ALT level 7 (20%) [28].

In addition to nonhematological toxicities, cytotoxic chemotherapy predictably suppresses the hematopoietic system and causes different grade hematological toxicities [29, 30]. As explained in our results, the majority of these hematological toxicities happened during the first four cycles of chemotherapy. The incidence of neutropenia increased from 44 (30%) on the 1^st^ cycle to 59 (40.4%) on the 4^th^ cycle. Likewise, the incidence of leukopenia increased from 38 (26%) to 68 (46.6%), and the incidence of anemia increased from 22 (15.1%) to 31 (21.2%) through the first four cycles. Moreover, the highest frequencies of grade 3 or 4 hematological toxicities were reported on 3^rd^ and 4^th^ cycles. Neutropenia (28 (19.2%)) was the most frequently reported grade ¾ hematological toxicities on the 4^th^ cycle. However, the incidence of grade 4 neutropenia in breast cancer subjects in five European countries (*n* = 444) was 34% [18]. In addition, one pooled analysis of RCTs reported neutropenia (31% overall, 28.2% grade ¾), anemia (30% overall, 1.3% grade ¾), and leucopenia (26% overall, 24.3% grade ¾) [23]. The incidences of these toxicities are relatively higher than our present finding. This is perhaps due to some of the patients in the study received higher dose intensity and dose-dense (i.e., more frequent) chemotherapy than our study participants.

Chemotherapy predisposes patients with cancer to infections both by suppressing the production of neutrophils and by its cytotoxic effects on the cells that line the alimentary tract [29, 31]. Conversely, a large number of our study participants were utilized with G-CSF 17 (11.6%) and oral ciprofloxacin 17 (11.6%) during the 3^rd^ cycle chemotherapy to prevent infection associated with neutropenia. This is supported by the fact that antibacterial prophylaxis with at least seven days of oral ciprofloxacin is recommended to prevent invasive infection by Gram-negative bacilli in outpatients with profound neutropenia and mucositis [30].

Fortunately, the incidences of hematological toxicities were declined during the last four cycles of chemotherapy in those patients who received 8 cycles of AC-T regimen. However, one patient during cycle 5 experienced grade 4 infections (i.e., meningitis); notably, this did not happen during the first four cycles. Surprisingly, no grade 3 infection and febrile neutropenia were documented in our study participants during the cohort. Contrary to our findings during the first four cycles, one large prospective cohort study in the USA revealed that the majority of neutropenic and infection events occurred in the first cycle and decreased in subsequent cycles in patients with solid tumors [32].

Myelosuppression, particularly neutropenia, in addition to predisposing to life-threatening infections, often leads to treatment delays and dose reductions that reduce the intensity of chemotherapy [33]. Consequently, identifying pretreatment and other predictor factors would be preferable for determining which patients are at greater risk. This enables caregivers to implement supportive measures before most complications would occur [29]. Therefore, we identified predictor factors associated with first cycle neutropenia, anemia, and others. As a result, having a lower baseline ANC (AOR = 0.670, *P*=0.002) was an independent predictor for neutropenia. Likewise, lower baseline Hgb (AOR = 0.339, *P* ≤ 0.001) and higher baseline ANC (AOR = 1.332, *P* ≤ 0.001) were significant predictors for anemia. In addition to having lower baseline Hgb (AOR = 0.700, *P*=0.016) and WBC (AOR = 0.856, *P*=0.047), being at a younger age (AOR = 0.964, *P*=0.021) was a significant predictor for vomiting. As it is noted from our results, lower baseline WBC, ANC, and Hgb were significant predictors of first cycle hematological toxicities incidences. The study by Crawford et al. [29] also revealed that age and pretreatment blood counts can predict chemotherapy-induced hematological toxicities.

In addition to decreasing neutropenic events, its duration, its severity, and its complication [9, 29, 34, 35], the use of G-CSF enables the patients to maintain standard RDI or optimum drug delivery [36–39]. As a result, 42 (28.8%) of our patients used G-CSF at least once during their treatment. Moreover, 61 (41.8%) of our study participants experienced dose delay at least once, with a median of seven days, with the most frequent G-CSF use (17 (11.6%)) and dose delay (31 (21.2%)) on cycle 3. This was because most frequent hematological toxicities happened on the 3^rd^ cycle. Indeed, it is related to cumulative dose bone marrow suppression of cyclophosphamide (C) and doxorubicin (A) [30].

Of 933 cycles delivered to our study participants, 95 (10%) cycles were delayed due to toxicities. Neutropenia was the main cause of the dose delay in this study. Of 95 cycles delayed due to toxicities, 89 (93.7%) cycles were delayed due to neutropenia, whereas anemia, infection, peripheral neuropathy, and oral mucositis attributed to dose delay in only 6 cycles. Different studies in different parts of the globe also revealed that neutropenia is the main dose-limiting toxicity in patients on chemotherapy [31, 40–42].

Delivering chemotherapy according to the plan (i.e., without dose delay) to breast cancer patients is critical in prolonging survival [43, 44]. A 20% dose reduction may compromise cure by 50%, and patients receiving less than 65% dose intensity are expected to have a survival similar to that of an untreated control group [45]. Gompertzian kinetics suggests that micrometastases in the adjuvant setting grow faster than established macrometastases; thus, there is higher regrowth of micrometastases between the cycles of chemotherapy. Therefore, the administration of cytotoxic drugs at least by conventional intensity or interval would be very important to minimize residual tumor burden [46].

Moreover, studies by Benadonna et al. and others revealed that breast cancer patients should receive at least 85% of their planned chemotherapy dose intensity to get benefit from chemotherapy [29, 47]. Nevertheless, the average relative dose intensity of doxorubicin, cyclophosphamide, and paclitaxel was above 85%, and we found that the significant number of patients received below the standard relative dose intensity (i.e., RDI < 85%). That is, 38/146 (26%) and 9/75 (12%) of our study participants received less than 85% of their planned dose intensity of doxorubicin and cyclophosphamide and paclitaxel, respectively. Considering these three agents together, 44 (30.1%) of our study participants received below the standard (i.e., RDI < 85%).

The seven-year data extracts of women with early breast cancer in the USA (*n* = 626) indicated that the incidence of dose delay and RDI < 85% for non-dose-dense AC was 24% and 17%, respectively [48]. The frequency of both dose delay and RDI < 85% is lower than that of our findings. These might be related to higher utilization of neutropenia preventive measures in the USA. However, the same study [48] reported that those who received both dose-dense and non-dose-dense AC-T regimen experienced more frequent dose delay (42% for dose-dense, 43% for non-dose-dense) and RDI < 85% (32% for dose-dense, 51% for non-dose-dense). These were related to AC-T regimen dose density and the number of cycles the patients received.

A survey of 190 community oncology practices between 1998 and 2002 with 3,707 early breast cancer subjects reported that average RDI for all regimens was 88%, in which 30% of the patients received <85% of the standard [49]. Similar results were reported in our study with average RDI of all drugs above 90% and overall RDI < 85% in 44 (30.1%) patients. However, Weycker et al. [48] reviewed that the incidence of RDI < 85% in women with breast cancer in USA between 1997 and 2000 was 59%. This higher incidence of RDI < 85% in USA happened though the majority of the subjects in the study received non-dose-dense (i.e., less frequent) doxorubicin- and cyclophosphamide- (AC-) containing regimen.

The range of RDI of Adriamycin and cyclophosphamide in Asian early-stage breast cancer patients (70–105%) [42] was higher than that reported in our patients (59–102%). One retrospective study from Canada also indicated that overall 14.4% of patients received less than 85% of planned chemotherapy intensity (i.e., FEC-100, FEC-D, and AC-T), considering only those received AC-T regimen, 96% of them had RDI ≥ 85% [13]. However, in our case, the frequency of those who received overall RDI ≥ 85% was 69.9%. This huge difference might be due to the higher utilization of G-CSF in Canadian breast cancer patients [13] since the use of G-CSF at least in part reduces the incidence of RDI < 85% [35, 48–50]. Moreover, all hematopoietic supports given to our patients were reactive or therapeutic and not prophylactic.

Different scholars identified treatment and patient-related factors associated with low RDI. Treatment-related factors are NE occurrence, higher stage of the disease, regimen type, concomitant radiotherapy administration, age, body surface area, body mass index, negative lymph node, comorbidity with renal impairment, under- or nonuse of granulocyte colony-stimulating factor (G-CSF), low-performance status, and anthracycline-based regimens. Patient-related factors may include appointment cancellations, patient noncompliance, patient knowledge deficits, and restricted access to care which are significantly associated with low RDI [9, 18, 31, 49, 51, 52].

Among the above-explained factors by different scholars, our study revealed that only moderate to higher grade neutropenia had a significant association (*P*=0.027) with RDI < 85% for doxorubicin and cyclophosphamide while it was not for paclitaxel (*P*=0.511) (data not shown). In addition to neutropenia, treatment intervals may become longer than the planned one because of patient's social factors or calendar conflicts [43, 53] which might be the cause for lower RDI for paclitaxel in our patients. However, overall, grade ¾ neutropenia (AOR = 0.26, *P*=0.001) and receiving 8 cycles (AOR = 0.28, *P*=0.006) were the two significant determinants that decrease the probability of having overall RDI ≥ 85% by 74% and 72% compared with their counterparts, respectively.

A study done in Korea reported lower dose delay (19.5%) [54] which attributed to higher utilization of G-CSF as both primary and secondary prophylaxis for neutropenia and its complication [28, 54]. Moreover, the incidence of dose delay in six retrospective European audits of breast cancer chemotherapy was 26% [31] which is lower than that of our result. The higher incidence of dose delay, which resulted in RDI < 85%, in our study participants is related to the higher incidence of neutropenia. However, there was low utilization of G-CSF to reduce the incidence of neutropenia. This was due to the majority of the present study participants could not afford the cost of G-CSF [42].

In general, the main goal of breast cancer chemotherapy is to increase disease-free survival and overall survival of the patients [1, 2] though it causes diverse aspects of adverse drug reactions [3, 4] that will deteriorate different domains of the patients' quality of life [19]. On the contrary, receiving less than optimum RDI (i.e., RDI < 85%) resulted in lower survival for those patients [29, 31, 47, 55]. Hence, in light of the well documented lower survival in patients who were treated with RDI < 85% [29, 31, 43–46, 55], in particular, due to neutropenia, an appropriate treatment or prophylactic G-CSF or antibiotic therapy should be given to the patients at the highest risk of neutropenia based on pretreatment ANC. Besides, educating patients about the importance of adhering to their treatment schedule [11, 12] has great importance.

### 4.1. Limitation of the Study

The study relatively had a small sample size. Moreover, this study was not designed for efficacy endpoints due to the short follow-up period to determine the impact of lower RDI on DFS and OS. We are also unable to differentiate those participants who are enrolled in a palliative care program. An additional limitation is that the response of the treating clinicians to the reported toxicities is unknown due to the patient-reported outcome nature of the study. It is also possible that the retrospective self-reporting of adverse effects at three-week intervals may have introduced recall bias into participant responses regarding subjective toxicities.

## 5. Conclusion

Ethiopian women with breast cancer on anthracycline-based AC and AC-T chemotherapy predominantly experienced grade 1 to 3 hematological and nonhematological ADRs, particularly during the first four cycles, in a routine clinical care setting. Neutropenia was the only toxicity that led to RDI < 85%. Hence, the utilization of G-CSF should be enhanced to decrease the incidence of reduced RDI below 85%. Pretreatment blood cell counts can be used to identify patients at increased risk of significant myelosuppression and vomiting at the start of chemotherapy.

## Figures and Tables

**Table 1:** Sociodemographic and clinical characteristics of women with breast cancer on AC and AC-T chemotherapy at TASH, from January 1 to September 30, 2017 GC, *N* = 146.

	Category	*N* (%)	Mean ± SD

Age (year)	20–34	30 (20.5)	42.2 ± 11.5
	35–49	76 (52.1)	
	50–64	31 (21.2)	
	≥65	9 (6.2)	

Body mass index (BMI) (Kg·m^−2^)	<18.5	14 (9.6)	25.2 ± 10.32
	18.5–24.99	74 (50.7)	
	25–29.99	30 (23.3)	
	≥30	24 (16.4)	

Body surface area (m^2^)	1–1.49	31 (21.2)	1.6 ± 0.19
	1.5–1.99	110 (75.3)	
	≥2	5 (3.4)	

Chemotherapy cycles	4 cycles	46 (31.5)	Median = 8 cycles
	6 cycles	25 (17.1)	
	8 cycles	75 (51.4)	

Histological classification	Ductal	131 (89.7)	
	Lobular	6 (4.1)	
	Mixed	3 (2.1)	
	Papillary	3 (2.1)	
	Mucinous	2 (1.4)	
	Metaplastic	1 (0.7)	

Stage	I	6 (4.1)	
	II	48 (32.8)	
	III	64 (43.8)	
	IV	28 (19.2)	

Comorbidity	Yes	22 (15.1)	
	No	124 (84.9)	

ECOG performance	0	3 (2.1)	
	I	135 (92.5)	
	II	5 (3.4)	
	III	3 (2.1)	

Marital status	Single	10 (6.7)	
	Married	98 (67.1)	
	Divorced/widowed	38 (26.2)	

Educational status	Illiterate	58 (39.7)	
	Semiliterate^*∗*^	63 (43.2)	
	Literate^*∗*^	25 (17.1)	

Baseline laboratory values	Mean ± SD		Normal range^*∗∗*^
SCr (mg/dl)	1.0 (0.18)		0.5–1.2
AST (U/L)	27.7 (21.87)		≤40
ALT (U/L)	23.5 (29.25)		≤40
ALP (U/L)	227.9 (212.11)		≤270
WBC (10^3^/mm^3^)	7.3 (2.40)		4–10
Hgb (gm/dL)	13.9 (1.36)		12–16
PLT (10^3^/mm^3^)	314.1 (110.58)		150–450
ANC (10^3^/mm^3^)	4.1 (1.97)		2–7.8
Lympho (10^3^/mm^3^)	2.4 (0.80)		1.2–3.4

^*∗*^Semiliterate = those who attended elementary/high school or grade 1–12^th^; literate = those who had a college diploma/degree; and others are considered illiterate. ^*∗∗*^Normal range is a reference range/value which is expected for healthy women. AC, Adriamycin-cyclophosphamide; AC ⟶ T, Adriamycin-cyclophosphamide ⟶ paclitaxel; ALP, alkaline phosphatase; ALT, alanine aminotransferase; ANC, absolute neutrophil count; AST, aspartate aminotransferase; BMI, body mass index; BSA, body surface area; ECOG, Eastern Cooperative Oncology Group; GC, Gregorian Calendar; Hgb, hemoglobin; Lympho, lymphocytes; PLT, platelet count; SCr, serum creatinine; SD, standard deviation; TASH, Tikur Anbessa Specialized Hospital; WBC, white blood cell count.

**Table 2:** Overall grade toxicity profile, the pattern of G-CSF use, and treatment delay among breast cancer patients who received AC and AC-T chemotherapy at TASH, from January 1 to September 30, 2017 GC.

Chemotherapy cycles								
Variables	1	2	3	4	5	6	7	8
*N* (%)	146 (%)	146 (%)	146 (%)	146 (%)	100 (%)	100 (%)	75 (%)	75 (%)

*Hematological toxicities*								
Neutropenia	44 (30)	57 (39)	57 (39)	59 (40.4)	18 (18)	13 (13)	10 (13.3)	5 (6.8)
Grade 3	20 (13.7)	9 (6.2)	22 (15.1)	23 (15.8)	6 (6)	1 (1)	2 (2.7)	—
Grade 4	3 (2.1)	6 (4.1)	5 (3.4)	5 (3.4)	2 (2)	2 (2)	—	—
*Leukopenia*	38 (26)	49 (34)	52 (35.6)	68 (46.6)	17 (17)	20 (20)	8 (10.7)	10 (13.5)
Grade 3	2 (1.4)	—	3 (2.1)	1 (0.7)	—	1 (1)	1 (1.3)	1 (1.3)
Grade 4	—	—	—	—	—	—	—	—
*Anemia*	22 (15.1)	24 (16)	29 (19.9)	31 (21.2)	17 (17)	13 (13)	10 (13.3)	9 (12.2)
Grade 3	1 (0.7)	—	2 (1.4)	—	—	—	—	—
Grade 4	—	—	—	—	—	—	—	—
*Lymphopenia*	27 (18.5)	29 (20)	56 (38.3)	46 (31.5)	28 (28)	21 (21)	9 (12)	12 (16.2)
Grade 3	1 (0.7)	—	2 (1.4)	2 (1.4)	2 (2)	2 (2)	—	—
Grade 4	—	—	—	—	—	—	—	—
*Thrombocytes * ^*∗*^	2 (1.4)	3 (2.1)	2 (1.4)	5 (3.4)	2 (2)	2 (2)	1 (1.3)	1 (1.3)
Grade 3	—	—	—	—	—	—	—	—
Grade 4	—	—	—	—	—	—	—	—

*Nonhematological toxicities*								
*AST* ↑	12 (8.2)	11 (7.5)	16 (11)	16 (11)	11 (11)	10 (10)	7 (9.3)	7 (9.5)
Grade 3	—	2 (1.4)	—	—	—	—	—	—
Grade 4	—	—	—	—	—	—	—	—
*ALT* ↑	7 (4.8)	4 (2.7)	5 (3.4)	8 (5.5)	9 (9)	9 (9)	7 (9.3)	7 (9.5)
Grade 3	2 (1.4)	—	—	—	—	1 (1)	—	—
Grade 4	—	—	—	—	—	—	—	—
*ALP* ↑	22 (15.1)	19 (13)	17 (11.6)	17 (11.6)	18 (18)	18 (18)	9 (12)	11 (14.9)
Grade 3	2 (1.4)	—	—	1 (0.7)	1 (1)	1 (1)	1 (1.3)	1 (1.3)
Grade 4	—	—	—	—	—	—	—	—
*Cr* ↑	11 (7.5)	9 (6.7)	13 (8.9)	8 (5.5)	3 (3)	5 (5)	4 (5.3)	2 (2.7)
Grade 3	—	—	—	—	—	—	—	—
Grade 4	—	—	—	—	—	—	—	—
*Infection*	1 (0.7)	2 (1.4)	6 (4.1)	0 (0)	2 (2)	2 (2)	4 (5.3)	0 (0)
Grade 3	—	—	—	—	—	—	—	—
Grade 4	—	—	—	—	1 (1)	—	—	—
*Nausea * ^##^	123 (84.3)	124 (85)	124 (84.9)	110 (75.3)	36 (36)	26 (26)	7 (9.3)	5 (6.8)
Grade 3	31 (21.2)	24 (16.4)	14 (9.6)	18 (12.3)	1 (1)	1 (1)	—	—
*Vomiting*	92 (63)	88 (60.3)	96 (65.8)	86 (58.9)	24 (24)	19 (19)	1 (1.3)	1 (1.3)
Grade 3	12 (8.2)	7 (4.8)	10 (6.8)	14 (9.6)	—	—	—	—
Grade 4	—	—	—	—	—	—	—	—
*OM*	41 (28.1)	53 (36.3)	52 (35.6)	50 (30.2)	14 (14)	17 (17)	8 (10.7)	8 (10.8)
Grade 3	5 (3.4)	8 (5.5)	6 (4.1)	9 (6.2)	2 (2)	2 (2)	—	—
Grade 4	—	—	—	—	—	—	—	—
*Fatigue * ^##^	136 (93.2)	135 (92.5)	134 (91.8)	125 (85.6)	83 (83)	79 (79)	51 (68)	49 (66.2)
Grade 3	3 (2.1)	1 (0.7)	5 (3.4)	6 (4.1)	3 (3)	—	2 (2.7)	2 (2.7)
*PNP*	32 (21.9)	44 (30.1)	43 (29.5)	51 (34.9)	66 (66)	67 (67)	64 (85.3)	63 (85.1)
Grade 3	—	—	1 (0.7)	—	16 (16)	15 (15)	15 (20)	14 (18.9)
Grade 4	—	—	—	—	—	—	—	—

*Supportive measures given*								
G-CSF use^*∗∗*^	9 (6.2)	9 (6.2)	17 (11.6)	15 (10.3)	5 (5)	2 (2)	2 (2.7)	0 (0)
Antibiotic^$^	9 (6.2)	9 (6.2)	17 (11.6)	15 (10.3)	5 (5)	2 (2)	2 (2.7)	0 (0)
T. Delay^#^	17 (11.6)	9 (6.2)	31 (21.2)	21 (14.4)	8 (8)	6 (6)	3 (4)	0 (0)

“—” = no grade 3 or 4 toxicity reported; grade 3 and grade 4 represent a severe and life-threatening form of adverse drug reactions, respectively. ^*∗*^Thrombocytopenia. ^*∗∗*^Granulocyte colony-stimulating factor use for different grade neutropenia. ^$^Antibiotic use was oral ciprofloxacin 500 mg twice a day for seven days. ^#^T.delay = treatment delay due to toxicity. OM = oral mucositis; PNP = peripheral neuropathy. ^##^Grade 4 and above are not available for nausea and fatigue on the PRO part of NCI CTCAE (version 4.03).

**Table 3:** Relative dose intensity of Adriamycin, cyclophosphamide, and paclitaxel among breast cancer patients on AC and AC-T regimen at TASH, from January 1 to September 30, 2017 GC.

Drug	Dosing	*N*	RDI (%)^*∗*^	Range	*N* (%) of patients with	
					RDI < 85%	Overall RDI ≥ 85%

A	60 mg/m^2^Q3W for 4 or 6 cycles	146	90.40 ± 8.876	59.57–101.61	38 (26)	102 (69.9)^*∗*^
C	600 mg/m^2^ Q3W for 4 or 6 cycles	146	90.40 ± 8.876	59.57–101.61	38 (26)	
T	175 mg/m^2^Q3W for 4 cycles	75	93.35 ± 8.592	53.50–102.44	9 (12)	

A = Adriamycin; C = cyclophosphamide; Q3W = every three weeks; RDI = relative dose intensity; T = paclitaxel ^*∗*^44 (30.1%) of the patients had overall RDI < 85%.

**Table 4:** Determinants of overall RDI ≥ 85% among breast cancer patients who received AC and AC-T chemotherapy at TASH, from January 1 to September 30, 2017 GC, *N* = 146.

Factors^*∗*^	*N* (%)	COR	95% CI	*P* value	AOR	95% CI	*P* value

Neutropenia^#^							
Grade 0–2	88 (60.3%)	1	—	—	1	—	—
Grade¾	58 (39.7%)	0.714	0.56–0.91	0.006	0.26	0.116–0.581	0.001

Number of cycles received							
Cycle 4	46 (31.5%)	1	—	—	1	—	—
Cycle 6	25 (17.1%)	1.277	0.35–4.66	0.711	1.58	0.415–5.995	0.504
Cycle 8	75 (51.4%)	0.345	0.146–0.817	0.016	0.28	0.11–0.695	0.006

Regimen							
AC	71 (48.6%)	1	—	—	—	—	—
AC-T	75 (51.4%)	0.318	0.149–0.678	0.003	—	—	NS

AOR = adjusted odds ratio; COR = crude odds ratio; NS = not significant ^*∗*^Other sociodemographic, pretreatment laboratory values or other clinical data explained in Table 1 and other toxicities explained in Table 2 above had no significant association with overall RDI ≥ 85% (using multivariate backward logistic regression). ^#^Considering the maximum grade neutropenia reported during the follow-up as the toxicity grade for that patient.

**Table 5:** Factors associated with the incidence of first cycle toxicity among study participants, *N* = 146.

Factors^*∗*^	Mean ± SD or *N* (%)	COR	*P* value for COR	AOR	95% CI	*P* value

Determinants of first cycle neutropenia						
Baseline WBC	7.3 ± 2.40	0.756	0.005	—	—	NS
Baseline ANC	4.1 ± 1.97	0.670	0.002	0.670	0.519–0.867	0.002

Determinants of first cycle leukopenia						
Baseline Hgb	13.9 ± 1.36	0.772	0.050	0.735	0.544–0.991	0.044
Baseline WBC	7.3 ± 2.40	0.712	0.002	0.718	0.583–0.885	0.002
Baseline ANC	4.1 ± 1.97	0.706	0.009	—	—	NS

Determinants of first cycle Anemia						
Baseline Hgb	13.9 ± 1.36	0.345	*P* ≤ 0.001	0.339	0.206–0.558	*P* ≤ 0.001
Baseline ANC	4.1 ± 1.97	1.416	0.002	1.332	1.015–1.749	*P* ≤ 0.001
Baseline platelet	314.1 ± 110.6	1.005	0.034	—	—	NS
Baseline ALP	227.9 ± 212.1	1.003	0.042	—	—	NS
Tumor stage						
Stage I and II	54 (37)	1	—	—	—	—
Stage III	62 (42)	4.08	0.037	—	—	NS
Stage IV	30 (21)	5.17	0.025	—	—	NS

Determinants of first cycle vomiting						
Age	42.2 ± 11.5	0.963	0.015	0.964	0.935–0.994	0.021
Baseline WBC	7.3 ± 2.40	0.862	0.049	0.856	0.734–0.998	0.047
Baseline Hgb	13.9 ± 1.36	0.739	0.033	0.700	0.524–0.934	0.016

ALP, alkaline phosphatase; ANC, absolute neutrophil count; Hgb, hemoglobin; SD, standard deviation; WBC, white blood cell count. The baseline represents the pretreatment value. ^*∗*^No association was found with other sociodemographic and clinical data explained in Table 1.

## Data Availability

The data used to support the findings of the study are available from the corresponding author upon request.

## Supplementary Materials

Supplementary Materials Supplementary material: National Cancer Institute Common Terminology Criteria for Adverse Events (CTCAE) Version 4.03 for safety/toxicity assessment.

## References

[1] Henderson I. C., Berry D. A., Demetri G. D. (2003). Improved outcomes from adding sequential paclitaxel but not from escalating doxorubicin dose in an adjuvant chemotherapy regimen for patients with node-positive primary breast cancer. *Journal of Clinical Oncology*.

[2] Mamounas E. P., Bryant J., Lembersky B. (2005). Paclitaxel after doxorubicin plus cyclophosphamide as adjuvant chemotherapy for node-positive breast cancer: results from NSABP B-28. *Journal of Clinical Oncology*.

[3] Pearce A., Haas M., Viney R. (2017). Incidence and severity of self-reported chemotherapy side effects in routine care: a prospective cohort study. *PLoS One*.

[4] Behera S. K., Kishtapati C. R., Gunaseelan V., Dubashi B., Chandrasekaran A., Selvarajan S. (2017). Chemotherapy induced adverse drug reactions in cancer patients in a tertiary care hospital in south India. *Journal of Young Pharmacists*.

[5] Fromme E. K., Eilers K. M., Mori M., Hsieh Y.-C., Beer T. M. (2004). How accurate is clinician reporting of chemotherapy adverse effects? A comparison with patient-reported symptoms from the quality-of-life questionnaire C30. *Journal of Clinical Oncology*.

[6] Maio M. D., Gallo C., Leighl N. B. (2015). Symptomatic toxicities experienced during anticancer Treatment : agreement between patient and physician reporting in three randomized trials. *Journal of Clinical Oncology*.

[7] Henry D. H., Viswanathan H. N., Elkin E. P., Traina S., Wade S., Cella D. (2008). Symptoms and treatment burden associated with cancer treatment: results from a cross-sectional national survey in the U.S. *Supportive Care in Cancer*.

[8] Kuo S.-H., Lien H.-C., You S.-L. (2008). Dose variation and regimen modification of adjuvant chemotherapy in daily practice affect survival of stage I-II and operable stage III Taiwanese breast cancer patients. *The Breast*.

[9] Wildiers H., Reiser M. (2011). Relative dose intensity of chemotherapy and its impact on outcomes in patients with early breast cancer or aggressive lymphoma. *Critical Reviews in Oncology/Hematology*.

[10] Link B. K., Budd G. T., Scott S. (2001). Delivering adjuvant chemotherapy to women with early-stage breast carcinoma. *Cancer*.

[11] Fauci J. M., Whitworth J. M., Schneider K. E. (2011). Prognostic significance of the relative dose intensity of chemotherapy in primary treatment of epithelial ovarian cancer. *Gynecologic Oncology*.

[12] Chirivella I., Bermejo B., Insa A. (2009). Optimal delivery of anthracycline-based chemotherapy in the adjuvant setting improves outcome of breast cancer patients. *Breast Cancer Research and Treatment*.

[13] Raza S., Welch S., Younus J. (2009). Relative dose intensity delivered to patients with early breast cancer : canadian experience. *Current Oncology*.

[14] Deshpande P., Sudeepthi B., Rajan S., Abdul Nazir C. (2011). Patient-reported outcomes: a new era in clinical research. *Perspectives in Clinical Research*.

[15] Basch E., Reeve B. B., Mitchell S. A. (2014). Development of the national cancer institute’s patient-reported outcomes version of the common Terminology criteria for adverse events (PRO-CTCAE). *JNCI Journal of the National Cancer Institute*.

[16] Department of Health and Human Services U.S. (2010). *Common Terminology Ctriteria for Advers Events v4.0*.

[17] Friese C. R., Harrison J. M., Janz N. K. (2017). Treatment-associated toxicities reported by patients with early-stage invasive breast cancer. *Cancer*.

[18] Pettengell R., Schwenkglenks M., Leonard R. (2008). Neutropenia occurrence and predictors of reduced chemotherapy delivery: results from the INC-EU prospective observational European neutropenia study. *Supportive Care in Cancer*.

[19] Husebø A. M. L., Dyrstad S. M., Mjaaland I., Søreide J. A., Bru E. (2014). Effects of scheduled exercise on cancer-related fatigue in women with early breast cancer. *The Scientific World Journal*.

[20] Ding W., Li Z., Wang C., Dai J., Ruan G., Tu C. (2018). Anthracycline versus nonanthracycline adjuvant therapy for early Breast Cancer. *Medicine (Baltimore)*.

[21] Bhosle J., Hall G. (2009). Principles of cancer treatment by chemotherapy. *Surgery (Oxford)*.

[22] Rašić A., Sofić A., Bešlija S., Rašić I., Hasanbegović B. (2019). Effects of adding taxane to anthracycline-based neoadjuvant chemotherapy in locally advanced breast cancer. *Medicinski Glasnik*.

[23] Ntellas P., Spathas N., Agelaki S., Zintzaras E., Saloustros E. (2019). Chemotherapy as adjuvant treatment for Breast Cancer : a pooled analysis of randomized controlled trials by the hellenic academy of oncology. *Oncotarget*.

[24] Jones S. E., Savin M. A., Holmes F. A. (2006). Phase III trial comparing doxorubicin plus cyclophosphamide with docetaxel plus cyclophosphamide as adjuvant therapy for operable breast cancer. *Journal of Clinical Oncology*.

[25] Gupta S., Goldstein L. J. (2010). Docetaxel with cyclophosphamide is associated with an overall survival benefit compared with doxorubicin and cyclophosphamide: 7 year follow-up of US oncology research trial 9735. *Breast Diseases: A Year Book Quarterly*.

[26] Watanabe T., Kuranami M., Inoue K. (2017). Comparison of an AC-taxane versus AC-free regimen and paclitaxel versus docetaxel in patients with lymph node-positive breast cancer: final results of the national surgical adjuvant study of breast cancer 02 trial, a randomized comparative phase 3 study. *Cancer*.

[27] Ferreira A. R., Metzger-Filho O., Sarmento R. M. B., Bines J. (2018). Neoadjuvant treatment of stage IIB/III triple negative breast cancer with cyclophosphamide, doxorubicin, and cisplatin (CAP regimen): a single arm, single center phase II study (GBECAM 2008/02). *Frontiers in Oncology*.

[28] Kim W. Y., Seo J. H., Son G. S., Lee J. B., Woo S. U., Bae J. W. (2011). Toxicities, dose reduction and delay of docetaxel and paclitaxel chemotherapy in breast cancer without distant metastases. *Journal of Cancer Research and Therapeutics*.

[29] Crawford J., Dale D. C., Lyman G. H. (2004). Chemotherapy-induced neutropenia. *Cancer*.

[30] Fontanella C., Bolzonello S., Lederer B., Aprile G. (2014). Management of breast cancer patients with chemotherapy-induced neutropenia or febrile neutropenia. *Breast Care*.

[31] Schwenkglenks M., Jackisch C., Constenla M. (2006). Neutropenic event risk and impaired chemotherapy delivery in six European audits of breast cancer treatment. *Supportive Care in Cancer*.

[32] Culakova E., Thota R., Poniewierski M. S. (2014). Patterns of chemotherapy-associated toxicity and supportive care in US oncology practice: a nationwide prospective cohort study. *Cancer Medicine*.

[33] Jenkins P., Freeman S. (2009). Pretreatment haematological laboratory values predict for excessive myelosuppression in patients receiving adjuvant FEC chemotherapy for breast cancer. *Annals of Oncology*.

[34] Ye X., Zhai Q., Wang Z., Du Q., Zhu B., Yu B. (2017). Neutropenic complications in Chinese patients with breast cancer in a real-world setting. *International Journal of Clinical and Experimental Pathology*.

[35] Ozer H., Mirtsching B., Rader M. (2007). Neutropenic events in community practices reduced by first and subsequent cycle pegfilgrastim use. *The Oncologist*.

[36] Morrison V. A., Wong M., Hershman D., Campos L. T., Ding B., Malin J. (2007). Observational study of the prevalence of febrile neutropenia in patients who received filgrastim or pegfilgrastim associated with 3-4 week chemotherapy regimens in community oncology practices. *Journal of Managed Care Pharmacy*.

[37] Aapro M., Crawford J., Kamioner D. (2010). Prophylaxis of chemotherapy-induced febrile neutropenia with granulocyte colony-stimulating factors: where are we now?. *Supportive Care in Cancer*.

[38] Chan A., Verma S., Loibl S. (2012). Reporting of myelotoxicity associated with emerging regimens for the treatment of selected solid tumors. *Critical Reviews in Oncology/Hematology*.

[39] Bennett C. L., Djulbegovic B., Norris L. B., Armitage J. O. (2013). Colony-stimulating factors for febrile neutropenia during cancer therapy. *New England Journal of Medicine*.

[40] Han Y., Yu Z., Wen S., Zhang B., Cao X., Wang X. (2012). Prognostic value of chemotherapy-induced neutropenia in early-stage breast cancer. *Breast Cancer Research and Treatment*.

[41] Tashkandi E., Yan M., Younus J. (2015). Real world experience with dose dense ac-paclitaxel: two canadian cancer centers’ experience. *Journal of Solid Tumors*.

[42] Chan A., Chen C., Chiang J., Tan S. H., Ng R. (2012). Incidence of febrile neutropenia among early-stage breast cancer patients receiving anthracycline-based chemotherapy. *Supportive Care in Cancer*.

[43] Walker M., Ni O. (2007). Neuroprotection during chemotherapy. *American Journal of Clinical Oncology*.

[44] Park S., Krishnan A., Lin C., Goldstein D., Friedlander M., Kiernan M. (2008). Mechanisms underlying chemotherapy-induced neurotoxicity and the potential for neuroprotective strategies. *Current Medicinal Chemistry*.

[45] Skipper H. E. (1971). Kinetics of mammary tumor cell growth and implications for therapy. *Cancer*.

[46] Norton L. (1988). A gompertzian model of human breast cancer growth. *Cancer Research*.

[47] Bonadonna G., Valagussa P., Moliterni A., Zambetti M., Brambilla C. (1995). Adjuvant Cyclophosphamide, methotrexate, and fluorouracil in node-positive breast cancer–the results of 20 years of follow-up. *New England Journal of Medicine*.

[48] Weycker D., Barron R., Edelsberg J., Kartashov A., Lyman G. H. (2012). Incidence of reduced chemotherapy relative dose intensity among women with early stage breast cancer in US clinical practice. *Breast Cancer Research and Treatment*.

[49] Shayne M., Crawford J., Dale D. C., Culakova E., Lyman G. H. (2006). Predictors of reduced dose intensity in patients with early-stage breast cancer receiving adjuvant chemotherapy. *Breast Cancer Research and Treatment*.

[50] Garg P., Rana F., Gupta R., Buzaianu E. M., Guthrie T. H. (2009). Predictors of toxicity and toxicity profile of adjuvant chemotherapy in elderly breast cancer patients. *The Breast Journal*.

[51] Lyman G. H., Dale D. C., Crawford J. (2003). Incidence and predictors of low dose-intensity in adjuvant Breast Cancer chemotherapy: a nationwide study of community practices. *Journal of Clinical Oncology*.

[52] Vavra K. L., Saadeh C. E., Rosen A. L., Uptigrove C. E., Srkalovic G. (2013). Improving the relative dose intensity of systemic chemotherapy in a community-based outpatient cancer center. *Journal of Oncology Practice*.

[53] Yamaguchi H., Hirakawa T., Inokuchi K. (2011). Importance of relative dose intensity in chemotherapy for diffuse large B-cell lymphoma. *Journal of Clinical and Experimental Hematopathology*.

[54] Kim C. G., Sohn J., Chon H. (2016). Breast cancer incidence of febrile neutropenia in Korean female breast cancer patients receiving preoperative or postoperative Doxorubicin/Cyclophosphamide followed by docetaxel chemotherapy. *Journal of Breast Cancer*.

[55] Loibl S., Skacel T., Nekljudova V. (2011). Evaluating the impact of relative total dose intensity (RTDI) on patients’ short and long-term outcome in taxane- and anthracycline-based chemotherapy of metastatic breast cancer- a pooled analysis. *BMC Cancer*.

