# Supplementary material for: Patterns of Anthracycline-Based Chemotherapy-Induced Adverse Drug Reactions and Their Impact on Relative Dose Intensity among Women with Breast Cancer in Ethiopia: A Prospective Observational Study
Source: J Oncol. 2020 Feb 21;2020:2636514. doi: 10.1155/2020/2636514 (PMC7054818; doi:10.1155/2020/2636514)
Supplement: Supplementary Materials — Supplementary material: National Cancer Institute Common Terminology Criteria for Adverse Events (CTCAE) Version 4.03 for safety/toxicity assessment. [file 2636514.f1.pdf]

Supplementary material:-National Cancer Institute Common Terminology Criteria for Adverse Events (CTCAE) Version 4.03: for safety/toxicity assessment

	Grade				
**Adverse Event**	1	2	3	4	5
Constipation	Occasional or intermittent symptoms; occasional use of stool softeners, laxatives, dietary modification, or enema	Persistent symptoms with regular use of laxatives or enemas; limiting instrumental ADL	Obstipation with manual evacuation indicated; limiting self-care ADL	Life-threatening consequences; urgent intervention indicated	Death
Diarrhea	Increase of <4 stools per day over baseline; mild increase in ostomy output compared to baseline	Increase of 4 - 6 stools per day over baseline; moderate increase in ostomy output compared to baseline	Increase of >=7 stools per day over baseline; incontinence; hospitalization indicated; severe increase in ostomy output compared to baseline; limiting self-care ADL	Life-threatening consequences; urgent intervention indicated	Death
Gastritis/stomatitis	Asymptomatic; clinical or diagnostic observations only; intervention not indicated	Symptomatic; altered GI function; medical intervention indicated	Severely altered eating or gastric function; TPN or hospitalization indicated	Life-threatening consequences; urgent operative intervention indicated	Death
Dysgeusia	Altered taste but no change in diet	Altered taste with change in diet (e.g., oral supplements); noxious or unpleasant taste; loss of taste	–	–	–
Oral Mucositis	Asymptomatic or mild symptoms; intervention not indicated	Moderate pain; not interfering with oral intake; modified diet indicated	Severe pain; interfering with oral intake	Life-threatening consequences; urgent intervention indicated	Death

Supplementary material:-National Cancer Institute Common Terminology Criteria for Adverse Events (CTCAE) Version 4.03: for safety/toxicity assessment

Anal mucositis	Asymptomatic or mild symptoms; intervention not indicated	Symptomatic; medical intervention indicated; limiting instrumental ADL	Severe symptoms; limiting self care ADL	Life-threatening consequences; urgent intervention indicated	Death
Nausea	Loss of appetite without alteration in eating habits	Oral intake decreased without significant weight loss, dehydration or malnutrition	Inadequate oral caloric or fluid intake; tube feeding, TPN, or hospitalization indicated	_	_

Vomiting	1 - 2 episodes (separated by 5 minutes) in 24 hrs	3 - 5 episodes (separated by 5 minutes) in 24 hrs	>=6 episodes (separated by 5 minutes) in 24 hrs; tube feeding, TPN or hospitalization indicated	Life-threatening consequences; urgent intervention indicated	Death
Skin hyperpigmentation	Hyperpigmentation covering <10% BSA; no psychosocial impact	Hyperpigmentation covering >10% BSA; associated psychosocial impact	_	_	_
Allergic reaction	Transient flushing or rash, drug fever <38 degrees C (<100.4 degrees F); intervention not indicated	Intervention or infusion interruption indicated; responds promptly to symptomatic treatment (e.g., antihistamines, NSAIDS, narcotics); prophylactic medications indicated for <=24 hrs	Prolonged (e.g., not rapidly responsive to symptomatic medication and/or brief interruption of infusion); recurrence of symptoms following initial improvement; hospitalization indicated for clinical sequelae (e.g., renal impairment, pulmonary infiltrates)	Life-threatening consequences; urgent intervention indicated	Death

Supplementary material:-National Cancer Institute Common Terminology Criteria for Adverse Events (CTCAE) Version 4.03: for safety/toxicity assessment

	camouflage	impact			
Fatigue	Fatigue relieved by rest	Fatigue not relieved by rest; limiting instrumental ADL	Fatigue not relieved by rest, limiting self care ADL	_	_
Peripheral motor neuropathy	Asymptomatic; clinical or diagnostic observations only; intervention not indicated	Moderate symptoms; limiting instrumental ADL	Severe symptoms; limiting self care ADL; assistive device indicated	Life-threatening consequences; urgent intervention indicated	Death
Peripheral sensory neuropathy	Asymptomatic; loss of deep tendon reflexes or paresthesia	Moderate symptoms; limiting instrumental ADL	Severe symptoms; limiting self care ADL	Life-threatening consequences; urgent intervention indicated	Death
Paresthesia	Mild symptoms	Moderate symptoms; limiting instrumental ADL	Severe symptoms; limiting self care ADL	_	_
Epistaxis	Mild symptoms; intervention not indicated	Moderate symptoms; medical intervention indicated (e.g., nasal packing, cauterization; topical vasoconstrictors)	Transfusion, radiologic, endoscopic, or operative intervention indicated (e.g., hemostasis of bleeding site)	Life-threatening consequences; urgent intervention indicated	Death

Supplementary material:-National Cancer Institute Common Terminology Criteria for Adverse Events (CTCAE) Version 4.03: for safety/toxicity assessment

Edema limbs	5 - 10% inter-limb discrepancy in volume or circumference at point of greatest visible difference; swelling or obscuration of anatomic architecture on close inspection	>10 - 30% inter-limb discrepancy in volume or circumference at point of greatest visible difference; readily apparent obscuration of anatomic architecture; obliteration of skin folds; readily apparent deviation from normal anatomic contour; limiting instrumental ADL	>30% inter-limb discrepancy in volume; gross deviation from normal anatomic contour; limiting self-care ADL		
Thromboembolic event	Venous thrombosis (e.g., superficial thrombosis)	Venous thrombosis (e.g., uncomplicated deep vein thrombosis), medical intervention indicated	Thrombosis (e.g., uncomplicated pulmonary embolism [venous], non-embolic cardiac mural [arterial] thrombus), medical intervention indicated	Life-threatening (e.g., pulmonary embolism, cerebrovascular event, arterial insufficiency); hemodynamic or neurologic instability; urgent intervention indicated	Death
Alanine aminotransferase increased	>ULN - 3.0 x ULN	>3.0 - 5.0 x ULN	>5.0 - 20.0 x ULN	>20.0 x ULN	_
Aspartate aminotransferase increased	>ULN - 3.0 x ULN	>3.0 - 5.0 x ULN	>5.0 - 20.0 x ULN	>20.0 x ULN	_
Creatinine increased	>1 - 1.5 x baseline; >ULN - 1.5 x ULN	>1.5 - 3.0 x baseline; >1.5 - 3.0 x ULN	>3.0 baseline; >3.0 - 6.0 x ULN	>6.0 x ULN	_
Alkaline phosphatase increased	>ULN - 2.5x ULN	>2.5 - 5.0 x ULN	>5.0 - 20.0 x ULN	>20.0 x ULN	_
Lymphocyte count decreased	<LLN - 800/mm3	<800 - 500/mm3	<500 - 200/mm3	<200/mm3	_

Supplementary material:-National Cancer Institute Common Terminology Criteria for Adverse Events (CTCAE) Version 4.03: for safety/toxicity assessment

Neutrophil count decreased	<LLN - 1500/mm3	<1500 - 1000/mm3	<1000 - 500/mm3	<500/mm3	_
Platelet count decreased	<LLN - 75,000/mm3	<75,000 - 50,000/mm3	<50,000 - 25,000/mm3	<25,000/mm3	_
White blood cell decreased	<LLN - 3000/mm3	<3000 - 2000/mm3	<2000 - 1000/mm3	<1000/mm3	_
Anemia	Hemoglobin (Hgb) <LLN - 10.0 g/dL; <LLN - 6.2 mmol/L; <LLN - 100 g/L	Hgb <10.0 - 8.0 g/dL; <6.2 - 4.9 mmol/L; <100 - 80g/L	Hgb <8.0 g/dL; <4.9 mmol/L; <80 g/L; transfusion indicated	Life-threatening consequences; urgent intervention indicated	Death
Febrile neutropenia	_	_	ANC <1000/mm3 with a single temperature of >38.3 degrees C (101 degrees F) or a sustained temperature of >=38 degrees C (100.4 degrees F) for more than one hour.	Life-threatening consequences; urgent intervention indicated	Death
Hypotension	Asymptomatic, intervention not indicated	Non-urgent medical intervention indicated	Medical intervention or hospitalization indicated	Life-threatening and urgent intervention indicated	Death
Palmar-plantar erythrodysesthesia syndrome	Minimal skin changes or dermatitis (e.g., erythema, edema, or hyperkeratosis) without pain	Skin changes (e.g., peeling, blisters, bleeding, edema, or hyperkeratosis) with pain; limiting instrumental ADL	Severe skin changes (e.g., peeling, blisters, bleeding, edema, or hyperkeratosis) with pain; limiting self care ADL	_	_
Nail discoloration	Asymptomatic; clinical or diagnostic observations only; intervention not indicated	_	_	_	_

Supplementary material:-National Cancer Institute Common Terminology Criteria for Adverse Events (CTCAE) Version 4.03: for safety/toxicity assessment

Lung infection	_	Moderate symptoms; oral intervention indicated (e.g., antibiotic, antifungal, antiviral)	IV antibiotic, antifungal, or antiviral intervention indicated; radiologic, endoscopic, or operative intervention indicated	Life-threatening consequences; urgent intervention indicated	Death
Meningitis	_	_	IV antibiotic, antifungal, or antiviral intervention indicated; radiologic or operative intervention indicated; focal neurologic deficit	Life-threatening consequences; urgent intervention indicated	Death
Skin infection	Localized, local intervention indicated	Oral intervention indicated (e.g., antibiotic, antifungal, antiviral)	IV antibiotic, antifungal, or antiviral intervention indicated; radiologic or operative intervention indicated	Life-threatening consequences; urgent intervention indicated	Death
Mucosal infection	Localized, local intervention indicated	Oral intervention indicated (e.g., antibiotic, antifungal, antiviral)	IV antibiotic, antifungal, or antiviral intervention indicated; radiologic or operative intervention indicated	Life-threatening consequences; urgent intervention indicated	Death
Upper gastrointestinal hemorrhage	Mild; intervention not indicated	Moderate symptoms; medical intervention or minor cauterization indicated	Transfusion, radiologic, endoscopic, or elective operative intervention indicated	Life-threatening consequences; urgent intervention indicated	Death
Soft tissue infection	_	Localized; local intervention indicated (e.g., topical antibiotic, antifungal, or antiviral)	IV antibiotic, antifungal, or antiviral intervention indicated; radiologic or operative intervention indicated	Life-threatening consequences; urgent intervention indicated	Death

Supplementary material:-National Cancer Institute Common Terminology Criteria for Adverse Events (CTCAE) Version 4.03: for safety/toxicity assessment

Upper respiratory infection	_	Moderate symptoms; oral intervention indicated (e.g., antibiotic, antifungal, antiviral)	IV antibiotic, antifungal, or antiviral intervention indicated; radiologic, endoscopic, or operative intervention indicated	Life-threatening consequences; urgent intervention indicated	Death
Tooth infection	_	Localized; local intervention indicated (e.g., topical antibiotic, antifungal, or antiviral)	IV antibiotic, antifungal, or antiviral intervention indicated; radiologic or operative intervention indicated	Life-threatening consequences; urgent intervention indicated	Death
Urinary tract infection	_	Localized; local intervention indicated (e.g., topical antibiotic, antifungal, or antiviral)	IV antibiotic, antifungal, or antiviral intervention indicated; radiologic or operative intervention indicated	Life-threatening consequences; urgent intervention indicated	Death
Dry mouth	Symptomatic (e.g., dry or thick saliva) without significant dietary alteration; unstimulated saliva flow >0.2 ml/min	Moderate symptoms; oral intake alterations (e.g., copious water, other lubricants, diet limited to purees and/or soft, moist foods); unstimulated saliva 0.1 to 0.2 ml/min	Inability to adequately aliment orally; tube feeding or TPN indicated; unstimulated saliva <0.1 ml/min	_	_
Myalgia	Mild pain	Moderate pain; limiting instrumental ADL	Severe pain; limiting self care ADL	_	_
Arthralgia	Mild pain	Moderate pain; limiting instrumental ADL	Severe pain; limiting self care ADL	_	_

ADL=activities of daily living; ANC=absolute neutrophil count; LLN=lower limit of normal; ULN=upper limit of normal; TPN = Total Parenteral Nutrition.
